# Chitinase 3‐like 1 overexpression aggravates hypoxia–reoxygenation injury in IEC‐6 cells by inhibiting the PI3K/AKT signalling pathway

**DOI:** 10.1113/EP091768

**Published:** 2024-10-31

**Authors:** Lei Mi, Jie Jin, Yingying Zhang, Ming Chen, JianLi Cui, Rui Chen, Xiao Zheng, Changqing Jing

**Affiliations:** ^1^ Department of Gastrointestinal Surgery The Affiliated Taian City Central Hospital of Qingdao University Tai'an China; ^2^ Department of Traditional Chinese Medicine The Affiliated Taian City Central Hospital of Qingdao University Tai'an China; ^3^ Department of Gastrointestinal Surgery Shandong Provincial Hospital Affiliated to Shandong First Medical University Jinan China

**Keywords:** CHI3L1, intestinal ischaemia–reperfusion, NF‐κB, Nrf2, nuclear translocation, PI3K/AKT

## Abstract

Intestinal ischaemia–reperfusion (I/R) is a common clinical pathology with high incidence and mortality rates. However, the mechanisms underlying intestinal I/R injury remain unclear. In this study, we investigated the role and mechanism of chitinase 3‐like 1 (CHI3L1) during intestinal I/R injury. Therefore, we analysed the expression levels of CHI3L1 in the intestinal tissue of an intestinal I/R rat model and explored its effects and mechanism in a hypoxia–reoxygenation (H/R) IEC‐6 cell model. We found that intestinal I/R injury elevated CHI3L1 levels in the serum, ileum and duodenum, whereas H/R enhanced CHI3L1 expression in IEC‐6 cells. The H/R‐induced inhibition of proliferation and apoptosis was alleviated by CHI3L1 knockdown and aggravated by CHI3L1 overexpression. In addition, CHI3L1 knockdown alleviated, and CHI3L1 overexpression aggravated, the H/R‐induced inflammatory response and oxidative stress. Mechanistically, CHI3L1 overexpression weakened the activation of the phosphoinositide 3‐kinase (PI3K)/AKT pathway, suppressed the nuclear translocation of Nrf2, and promoted the nuclear translocation of nuclear factor κB (NF‐κB). Moreover, CHI3L1 knockdown had the opposite effect on the PI3K/AKT pathway, Nrf2, and NF‐κB. Moreover, the PI3K inhibitor LY294002 blocked the effect of CHI3L1 knockdown on the H/R‐induced inhibition of proliferation, apoptosis, inflammatory response and oxidative stress. In conclusion, CHI3L1 expression was induced during intestinal I/R and H/R injury in IEC‐6 cells, and CHI3L1 overexpression aggravated H/R injury in IEC‐6 cells by inhibiting the PI3K/AKT signalling pathway. Therefore, CHI3L1 may be an effective target for controlling intestinal I/R injury.

## Abstract (highlights)

- **What is the central question of this study?**
  Is CHI3L1 involved in intestinal ischaemia–reperfusion (I/R) injury?
- **What is the main finding and its importance?**
  Intestinal I/R injury elevated CHI3L1 levels in the ileum and duodenum. hypoxia–reoxygenation (H/R) enhanced CHI3L1 expression in IEC‐6 cells, and CHI3L1 overexpression aggravated, and knockdown alleviated, H/R injury in IEC‐6 cells. CHI3L1 may play a role in inducing intestinal I/R injury by inhibiting the phosphoinositide 3‐kinase/AKT signalling pathway. These findings provide evidence that findings provide evidence that the inhibition of CHI3L1 may be an effective way to alleviate intestinal I/R injury.

## 1 INTRODUCTION

Intestinal ischaemia–reperfusion (I/R) is a common clinical pathology with high incidence and mortality rates (Jin et al., 2022). Intestinal tissue ischaemia can be caused by pathological conditions, such as acute mesenteric ischaemia and haemorrhagic shock, or by surgical procedures, such as small bowel transplantation and cardiopulmonary bypass (Deng et al., 2021; Jin et al., 2022). The treatment of intestinal tissue ischaemia requires the restoration of blood flow, that is, reperfusion. However, reperfusion often leads to the production of reactive oxygen species (ROS) and the activation of inflammatory responses, exacerbating intestinal tissue damage (Li, Wang, et al., 2021). Intestinal I/R injury can lead to remote organ damage and heart, respiratory, liver or kidney failure. However, the mechanisms underlying the intestinal I/R injury remain unclear. Therefore, investigating the mechanism of intestinal I/R injury is essential for preventing and treating this injury and improving patient outcomes.

Chitinase 3‐like 1 (CHI3L1) is a glycoprotein member of the glycosyl hydrolase 18 family. It lacks chitinase activity and is secreted by macrophages, neutrophils, chondrocytes, smooth muscle cells, endothelial cells and tumour cells (Zhao et al., 2020). CHI3L1 plays an important role in oncogenesis, asthma, lung fibrosis, chronic obstructive pulmonary disease, liver fibrosis, bowel disease, atherosclerosis, coronary artery disease, diabetes, Alzheimer's disease and Parkinson's disease (Deutschmann et al., 2019; Zhao et al., 2020). Elevated serum CHI3L1 levels in patients with various diseases are associated with adverse outcomes (Zhao et al., 2020). The downstream regulation mechanism of CHI3L1 is complex, involving multiple signalling pathways, such as the c‐Jun N‐terminal kinase (JNK) pathway (Hong et al., 2023), transforming growth factor β pathway (Qiu et al., 2018), interleukin 13 receptor subunit α2 (IL‐13Rα2)/mitogen‐activated protein kinase pathway (Li, Wei, et al., 2021) and phosphoinositide 3‐kinase (PI3K)/AKT pathway (Wang et al., 2018).

There are a few reports on the role of CHI3L1 in regulating I/R injury. The present study suggests that blocking the upregulation of CHI3L1 can alleviate I/R injury in the liver (Jin et al., 2023), brain (Im et al., 2020) and kidneys (Montgomery et al., 2017). However, its role and mechanism of action in intestinal I/R injury remain unclear. Nevertheless, CHI3L1 is highly expressed in the intestinal mucosa of patients with inflammatory bowel disease and can be used as a serum marker of this disorder (Ning et al., 2019). Similar to inflammatory bowel disease, intestinal I/R injury activates inflammatory responses. Therefore, we hypothesized that CHI3L1 plays a role in the regulation of intestinal I/R injury.

In this study, we investigated the role and mechanism of action of CHI3L1 in the regulation of intestinal I/R injury. We analysed the expression of CHI3L1 in the intestinal tissue of an intestinal I/R rat model and explored its effects and mechanisms in a hypoxia–reoxygenation (H/R) cell model.

## 2 METHODS

### 2.1 Ethical approval

All animal experimental procedures were approved by the Medical Ethics Committee of Tai'an City Central Hospital (approval number: 2023‐07‐02) and conducted by the National Institutes of Health *Guide for the Care and Use of Laboratory Animals* (China). Ten male Sprague–Dawley (SD) rats of specific pathogen‐free grade, weighing 250−280 g, were purchased from Shanghai Jihui Experimental Animal Breeding (license number SCXK (Shanghai) 2022‐0009). All rats were housed under controlled conditions, maintained at a constant temperature of 20−26°C, humidity ranging from 50% to 60%, and a light–dark cycle of 12 h each. Ventilation was ensured by exchanging air 15−20 times per hour.

### 2.2 I/R model

Ten SD rats were randomly divided into two groups: a sham operation group (*n* = 5) and an intestinal I/R group (*n* = 5). After a 12‐h fasting period with unrestricted access to water, all the rats were intraperitoneally injected with 50 mg/kg sodium pentobarbital. The protocol for intestinal I/R was the same as that used in our previous study (Mi et al., 2021). The intestines were reperfused after 1 h of ischaemia. After 1 h of reperfusion, abdominal aortic blood was collected. All rats were euthanized and the duodenum and ileum were collected.

### 2.3 Histopathological analysis

Morphological changes in the ileum and duodenum of each group (*n* = 5) were observed by haematoxylin and eosin (HE) staining (Mi et al., 2021). The slides were scanned using a KF‐PRO‐120 Digital Pathology Slide Scanner (Ningbo Konfoong Bioinformation Tech Co., Ltd, Ningbo, China), and images were captured randomly. HE‐stained images were reviewed by senior pathologists and scores were assigned as follows: score 0, normal morphology, with small intestinal villi neatly arranged and devoid of inflammation; score 1, small intestinal villi irregularly arranged, with slight structural damage and mild inflammatory infiltration; score 2, villi tips showing damage, along with moderate inflammatory infiltration; score 3, extensive shedding of villi; moderate to severe inflammatory infiltration in the lamina propria, accompanied by a small amount of separation between the mucosal epithelium and the lamina propria; and score 4, significant structural damage, lamina propria rupture, and widespread inflammatory.

### 2.4 Immunohistochemistry

The CHI3L1 expression levels in the ileum and duodenum of each group (*n* = 5) were determined using immunohistochemistry. Briefly, dewaxing, hydration and antigen retrieval used the same method described in our previous study (Mi et al., 2021), in which 1:100 diluted anti‐CHI3L1 antibody (DF6577, Affinity Bioscience, Cincinnati, OH, USA) was added onto slides and incubated overnight at 4°C. After incubation for 1 h at room temperature, the slides were rinsed with phosphate‐buffered saline (PBS). Subsequently, a secondary antibody, goat anti‐rabbit IgG H&L (horseradish peroxidase (HRP) conjugated) (cat. no. ab97080, Abcam, Waltham, MA, USA), was added and incubated at 37°C for 30 min. Next, the slides were incubated with 3,3′‐diaminobenzidine substrate until the desired colour was achieved. Finally, the slides were stained with haematoxylin, rinsed and air‐dried before being coverslipped. The slides were scanned using a KF‐PRO‐120 Digital Pathology Slide Scanner (Ningbo Konfoong Bioinformation Tech Co.), and images were captured randomly. The expression level of CHI3L1 in each image was assessed by integrated optical density measured using Image Pro‐Plus 6.0 software (Media Cybernetics, Rockville, MD, USA).

### 2.5 Measurement of CHI3L1, interleukin‐1β, interleukin‐6 and malondialdehyde levels and superoxide dismutase activity

CHI3L1 levels in the IEC‐6 cell culture medium (*n* = 3) and rat serum (*n* = 5) were measured using a rat CHI3L1 ELISA kit (orb410170; Biorbyt, Cambridge, UK). Interleukin (IL)‐1β, IL‐6, and malondialdehyde (MDA) levels and superoxide dismutase (SOD) activity in rat serum (*n* = 5) and IEC‐6 cell culture medium (*n* = 3) were measured using rat IL‐1β enzyme‐linked immunosorbent assay (ELISA) kit (E‐EL‐R0012, Elabscience, Wuhan, China), rat IL‐6 ELISA kit (E‐EL‐R0015, Elabscience), MDA ELISA kit (E‐EL‐0060, Elabscience), and total SOD activity assay kit (E‐BC‐K020‐M, Elabscience), respectively.

### 2.6 Small interfering RNA and construction of CHI3L1 overexpression plasmid

Two small interfering RNAs (siRNAs) targeting CHI3L1 (CHI3L1‐si‐1 and CHI3L1‐si‐2) and a negative control siRNA (NC‐si) were purchased from Guangzhou RiboBio (Guangzhou, China). The target sequence of CHI3L1‐si‐1 and CHI3L1‐si‐3 was GGACCATACTAATTATACC and TGAAGTACCTGAAGAACAA, respectively. To construct the CHI3L1 overexpression plasmid, the complete coding sequence (CDS) of rat CHI3L1 isoform 1 (NM_001309820.1) was cloned into a pcDNA3.1+ plasmid. The primer sequences used to amplify the CDS of CHI3L1 by PCR were 5′‐cgcggatccgccaccATGTGCACCTCTGCTGAAGCCAGGATGGGC‐3′ (restriction enzyme cutting site: *Bam*HI) and 5′‐ccgctcgagCTAAGCCACAGCCAGGGCCTCCTTGATGGCGT‐3′ (restriction enzyme cutting site: *Xho*I). The CHI3L1‐overexpression plasmid and empty pcDNA3.1+ plasmid are referred to as CHI3L1‐OE and empty plasmids, respectively.

### 2.7 Cell culture and treatment

The rat intestinal epithelioid cell line no. 6 (IEC‐6) was purchased from Shanghai Anwei Biotechnology (Shanghai, China). The culture medium was Dulbecco's modified Eagle's medium, including 1.5 g/L NaHCO_3_ +10% fetal bovine serum, 1% penicillin–streptomycin, and 0.01 mg/mL insulin. The normoxia culture condition was 37°C and 95% air–5% CO_2_. The hypoxia culture condition was 37°C and 1% O_2_–5% CO_2_–94% N_2_. The final concentration of the PI3K inhibitor LY294002 was 10 µM.

#### 2.7.1 5‐Ethynyl‐2′‐deoxyuridine cell proliferation assay

After reoxygenation for 6 h, an 5‐ethynyl‐2′‐deoxyuridine (EdU) cell proliferation assay was performed using a C10310‐1 Cell‐LightTM EdU Apollo567 In Vitro Kit (Guangzhou Ribobio). Three biological replicates were used for each experiment.

### 2.8 Cell counting Kit‐8 assay

After reoxygenation for 24, 48 and 72 h, the optical density of each group of cells was measured at 450 nm using Cell Counting Kit‐8 (Dojindo, Kumamoto, Japan). Three biological replicates were used, each with three technical replicates.

### 2.9 Hoechst 33258 staining for apoptosis

After reoxygenation for 6 h, cells were rinsed twice with PBS and stained with Hoechst 33258 (Kasibhatla et al., 2006) staining reagent (Biosharp, Hefei, China) in the dark for 30 min. After washing twice with PBS, fluorescence was observed directly under a fluorescence microscope. Three biological replicates were used for each experiment.

### 2.10 Western blotting

After reoxygenation for 6 h, cells were harvested for western blot analysis. Nuclear and cytoplasmic proteins were extracted with the NE‐PER Nuclear and Cytoplasmic Extraction kit following the manufacturer's instructions (Thermo Fisher Scientific, Waltham MA, USA). Total protein isolation, protein concentration quantification, SDS‐PAGE and protein transfer were performed as described previously (Mi et al., 2021). After blocking with 5% skim milk powder in Tris‐buffered saline (TBS), membranes were incubated with primary antibody (anti‐CHI3L1 antibody, DF6577, Affinity Bioscience, anti‐nuclear factor κB (NF‐κB) p65 antibody, 1:1000, cat. no. 4764, Cell Signaling Technology, Danvers, MA, USA; anti‐Nrf2 antibody, 1:2000, ab313825, Abcam; anti‐AKT1 antibody, 1:1000, cat. no. 2938, Cell Signaling Technology; anti‐phosphorylated (p)‐AKT1 antibody, 1:1000, cat. no. 9018, Cell Signaling Technology; anti‐glyceraldehyde 3‐phosphate dehydrogenase (GAPDH) antibody, 1:5000, KC‐5G5, Aksomics, Shanghai, China; anti‐histone H3 antibody, 1:1000, cat. no. 14269, Cell Signaling Technology) at 4°C overnight. After rinsing with TBS containing 0.1% Tween 20 (TBST), the membranes were incubated with a HRP‐conjugated secondary antibody. After rinsing with TBST, the membranes were incubated with enhanced chemiluminescence in the dark and the luminescence intensity was visualized by exposing the signal to X‐rays. Three biological replicates were used for each experiment.

### 2.11 Statistical analysis

Data analysis was performed using SPSS 16.0 statistical software (SPSS Inc., Chicago, IL, USA). Student's *t*‐test was used to compare the two groups. One‐way analysis of variance was used for more than two groups, followed by a pairwise comparison between groups using Tukey's test. All data are expressed as means ± standard deviation, and differences were considered statistically significant at *P* < 0.05.

## 3 RESULTS

### 3.1 Intestinal I/R can elevate CHI3L1

**FIGURE 1:**
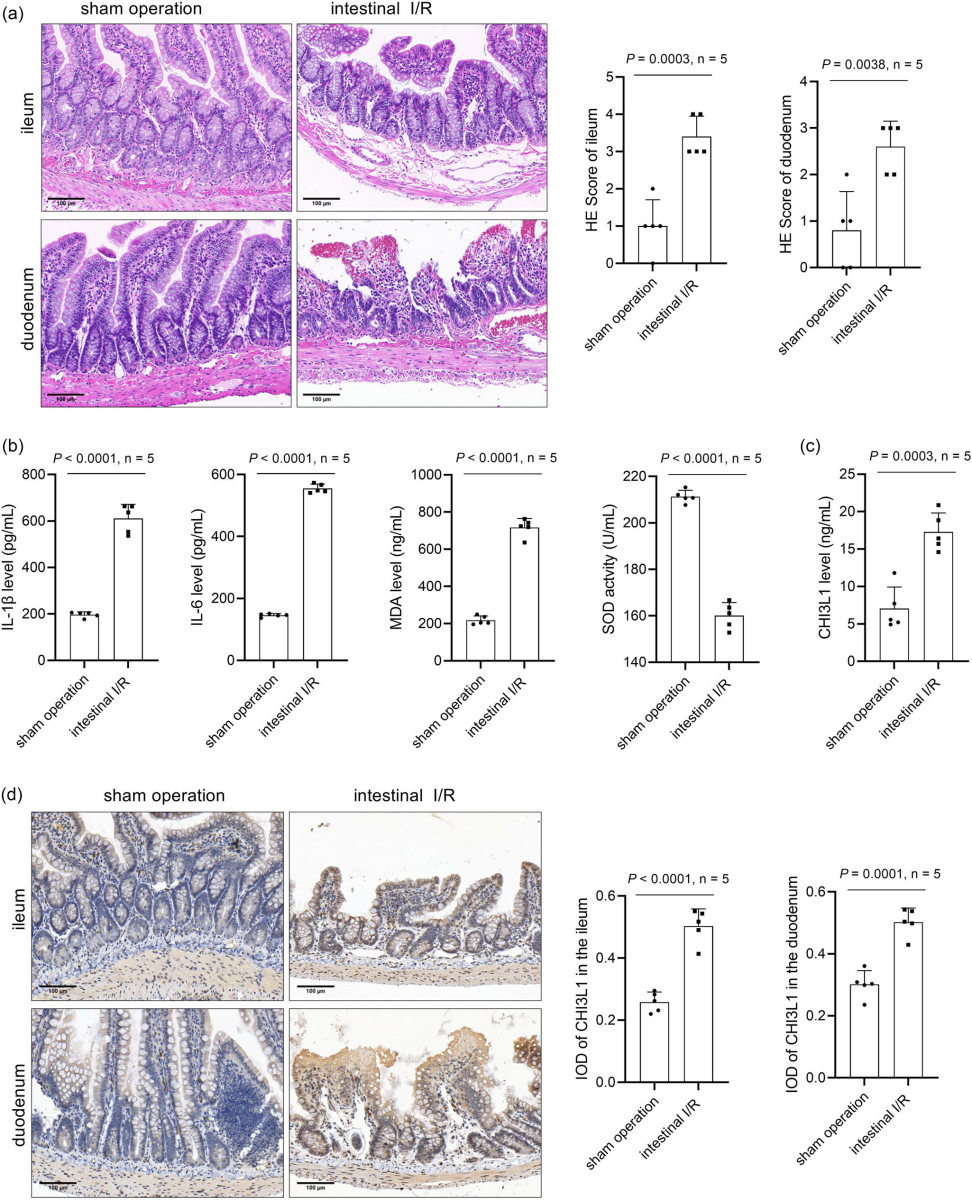
CHI3L1 expression level in sham operation and intestinal I/R rat models. (a) HE staining showing the morphological changes. (b) IL‐1β, IL‐6 and MDA levels and SOD activity in serum detected by commercial kits. (c) CHI3L1 level in serum detected using ELISA. (d) Immunohistochemistry results showing CHI3L1 expression level in the ileum and duodenum. Five data points were overlaid on the bar graph. For all groups, *n* = 5.

**FIGURE 2:**
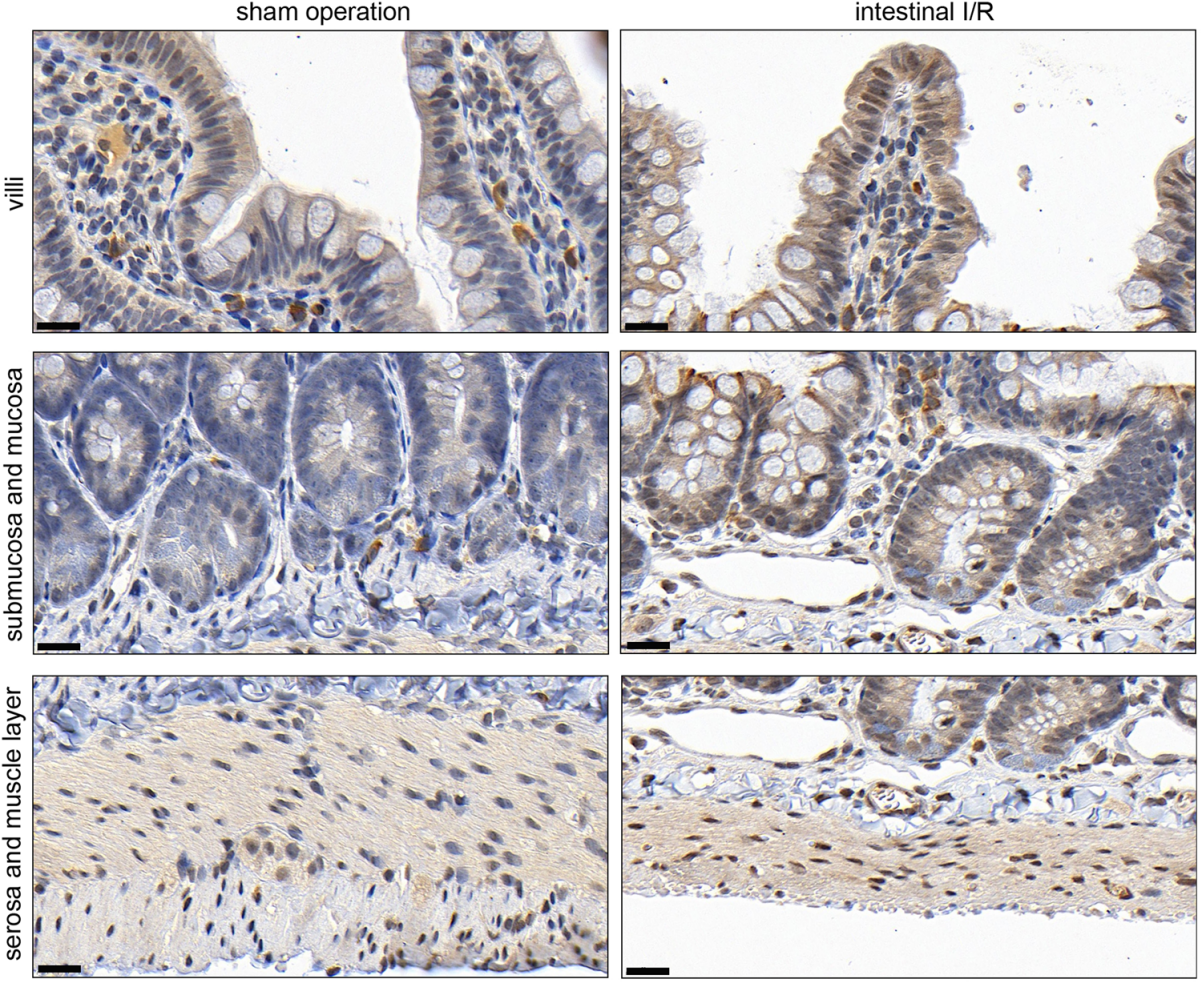
CHI3L1 levels in villi, mucosa, submucosa, the muscle layer and the serosa of the ileum of sham operation and intestinal I/R rat models. CHI3L1 levels were determined by immunohistochemistry. The scale bar indicates 20 µm.

**FIGURE 3:**
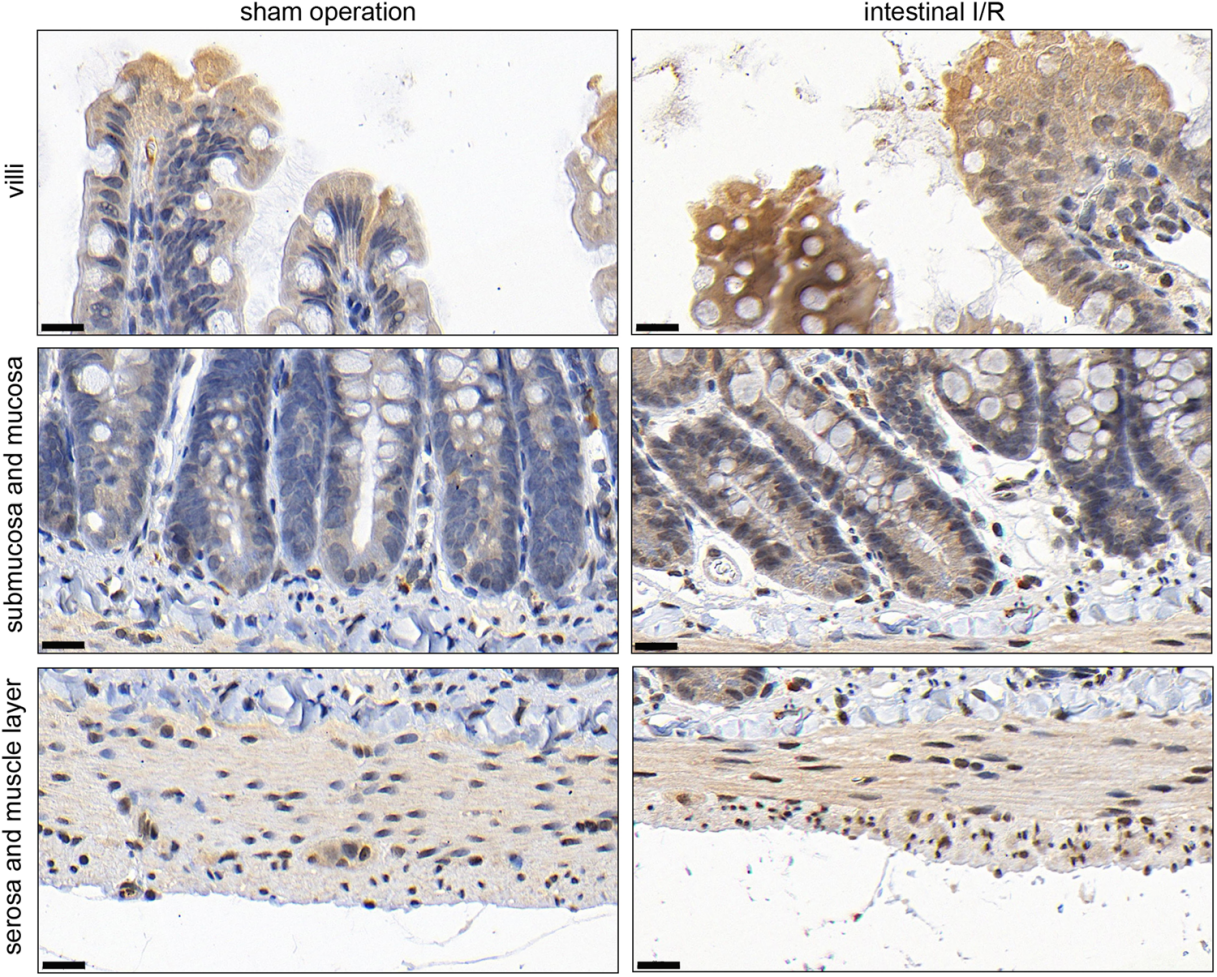
CHI3L1 levels in villi, mucosa, submucosa, the muscle layer and the serosa of the duodenum of sham operation and intestinal I/R rat models. CHI3L1 levels were determined by immunohistochemistry. The scale bar indicates 20 µm.

To confirm whether the intestinal I/R rat model was successfully constructed, we measured the morphological changes in the ileum and duodenum, and the levels of IL‐1β, IL‐6 and MDA in serum, and the SOD activity in serum. The results of the pathological analysis showed that the HE scores of the ileum (*P* = 0.0003) and duodenum (*P* = 0.0038) were both higher in the intestinal I/R group than in the sham operation group, indicating obvious structural damage to the ileum and duodenum of the intestinal I/R rat model (Figure 1a). The levels of IL‐1β (*P* < 0.0001), IL‐6 (*P* < 0.0001) and MDA (*P* < 0.0001) were increased, and SOD activity (*P* < 0.0001) was decreased in the intestinal I/R group compared to those in the sham operation group (Figure 1b). These results supported the successful establishment of an intestinal I/R rat model. Moreover, we found that CHI3L1 levels in the serum of the intestinal I/R rat model were higher than those in the serum of the sham operation group (*P* = 0.0003) (Figure 1c). In addition, immunohistochemistry showed that the integrated optical density of CHI3L1 in the ileum (*P* < 0.0001) and duodenum (*P* = 0.0001) of the intestinal I/R rat model was higher than that in the sham operation group (Figure 1d), indicating that intestinal I/R can elevate CHI3L1 levels in the ileum and duodenum. Magnified views of CHI3L1 staining in different intestinal cell types of the ileum and duodenum are shown in Figures 2 and 3.

### 3.2 H/R enhanced CHI3L1 expression in IEC‐6 cells

**FIGURE 4:**
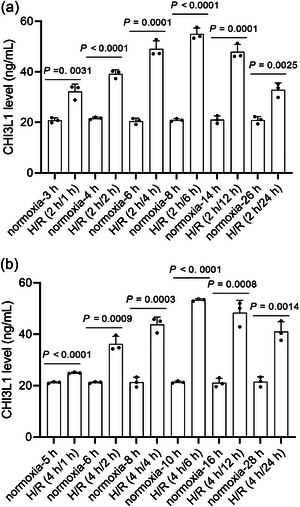
CHI3L1 level in culture medium after different hypoxia and reoxygenation (H/R) time detected using ELISA. Three data points were overlaid on the bar graph. For all groups, *n* = 3.

**FIGURE 5:**
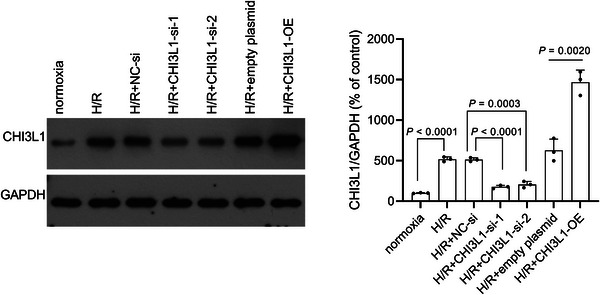
CHI3L1 level in different groups detected using western blotting. CHI3L1‐si‐1 and CHI3L1‐si‐2 were transfected into IEC‐6 cells to knock down CHI3L1 and NC‐si was transfected as a control. CHI3L1‐OE was transfected into IEC‐6 cells to overexpress CHI3L1. After transfection, IEC‐6 cells were cultured under hypoxia for 2 h and reoxygenated for 6 h (H/R). Cells cultured under normoxia were used as a control. Three data points were overlaid on the bar graph. For all groups, *n* = 3.

To construct the H/R IEC‐6 cell model, we measured the CHI3L1 levels in the culture medium at different hypoxia and reoxygenation times. Cells cultured under normoxic conditions were used as controls. After reoxygenation, the CHI3L1 content in the culture medium gradually increased, peaked at 6 h, and then gradually decreased (Figure 4a,b). Until 24 h after reoxygenation, CHI3L1 levels in the H/R group were higher than those in the normoxic group (all *P* < 0.01) (Figure 4a,b). Prolonged hypoxia from 2 to 4 h did not increase the secretion of CHI3L1 but slightly decreased it. Therefore, 2 h of hypoxia was used in subsequent assays. We also measured CHI3L1 levels in IEC‐6 cells from the normoxic and H/R groups by western blotting. As shown in Figure 5, the CHI3L1 levels were higher in the H/R group than in the normoxic group (*P* < 0.0001).

### 3.3 Effect of CHI3L1 on cell proliferation and apoptosis in IEC‐6 cells

**FIGURE 6:**
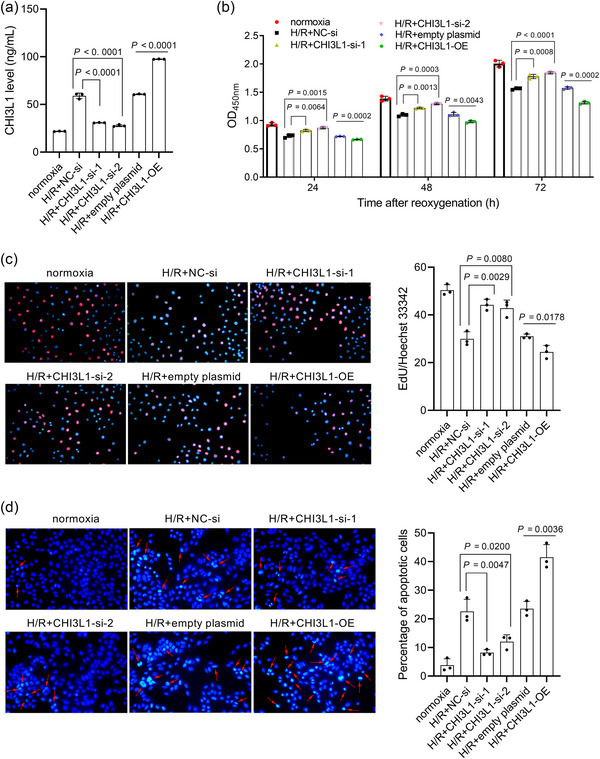
CHI3L1 knockdown alleviated and CHI3L1 overexpression aggravated H/R‐induced proliferation inhibition and apoptosis. CHI3L1‐si‐1 and CHI3L1‐si‐2 were transfected into IEC‐6 cells to knock down CHI3L1. CHI3L1‐OE was transfected into IEC‐6 cells to overexpress CHI3L1. After transfection, IEC‐6 cells were cultured under hypoxia for 2 h and reoxygenated for 6 h (H/R). Cells cultured under normoxia were used as a control. (a) CHI3L1 level in culture medium detected using ELISA. (b) The OD_450nm_ value of cells after continued culture for 24, 48 and 72 h after reoxygenation, detected using Cell Counting Kit‐8 assays. (c) EdU cell proliferation assay. Red is EdU‐positive cells; nuclei are blue. magnification: 20×. Right panel, bar graph of percentage of EdU‐positive cells. (d) Hoechst 33258 staining. Nuclei were stained by Hoechst 33258 and appeared blue. Apoptotic cells appear white and are indicated using arrows. magnification: 20×. Right panel, bar graph of the percentage of apoptotic cells. Three data points were overlaid on the bar graph. For all group, *n* = 3.

To investigate the regulatory role of CHI3L1 in intestinal I/R through assays in vitro, CHI3L1 was knocked down or overexpressed in H/R IEC‐6 cells to investigate its regulatory role in intestinal I/R. To avoid off‐target effects, two siRNAs, CHI3L1‐si‐1 and CHI3L1‐si‐2, were used to disrupt CHI3L1 expression. As shown in Figures 5 and 6a, CHI3L1 levels decreased after transfection with CHI3L1‐si‐1 (mRNA: *P* < 0.0001; protein: *P* < 0.0001) and CHI3L1‐si‐2 (mRNA: *P* < 0.0001; protein: *P* = 0.0003), and increased after transfection with CHI3L1‐OE (mRNA: *P* < 0.0001; protein: *P* = 0.0020), indicating that CHI3L1 was successfully knocked down or overexpressed. Subsequently, we found that IEC‐6 cells in the H/R+CHI3L1‐si‐1 and H/R+CHI3L1‐si‐2 groups had a stronger proliferative capacity than H/R+NC‐si, as indicated by the high OD_450nm_ value (all *P* < 0.01) (Figure 6b) and a high proportion of EdU‐positive cells (all *P* < 0.01) (Figure 6c). In addition, IEC‐6 cells in the H/R+CHI3L1‐OE group had a lower OD_450nm_ value (all *P* < 0.01) (Figure 6b) and a lower proportion of EdU‐positive cells than those in the H/R+empty plasmid group (all *P* < 0.05), suggesting that CHI3L1 overexpression aggravates the suppressive effect of H/R on the proliferative capacity of IEC‐6 cells. Cells were stained with Hoechst 33258 to evaluate apoptosis. The apoptotic cells are indicated by arrows in Figure 6d. IEC‐6 cells in the H/R+CHI3L1‐si‐1 (*P* = 0.0047) and H/R+CHI3L1‐si‐2 (*P* = 0.0200) groups had fewer apoptotic cells than those in the H/R+NC‐si group, while IEC‐6 cells in the H/R+CHI3L1‐OE group (*P* = 0.0036) had more apoptotic cells than those in the H/R+empty plasmid group. These results showed that the H/R‐induced inhibition of proliferation and apoptosis could be alleviated by CHI3L1 knockdown and aggravated by CHI3L1 overexpression.

### 3.4 Effect of CHI3L1 on inflammation and oxidative stress in IEC‐6 cells

**FIGURE 7:**
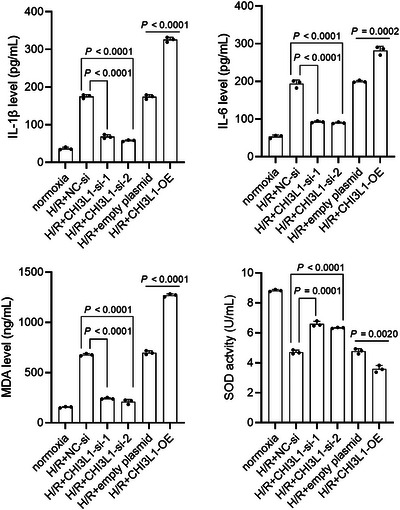
Effect of CHI3L1 knockdown and CHI3L1 overexpression on IL‐1β, IL‐6 and MDA levels and SOD activity in IEC‐6 cells. CHI3L1‐si‐1 and CHI3L1‐si‐2 were transfected into IEC‐6 cells to knock down CHI3L1. CHI3L1‐OE was transfected into IEC‐6 cells to overexpress CHI3L1. After transfection, IEC‐6 cells were cultured under hypoxia for 2 h and reoxygenated for 6 h (H/R). Cells cultured under normoxia were used as a control. IL‐1β, IL‐6 and MDA levels and SOD activity were detected by commercial kits. Three data points were overlaid on the bar graph. For all groups, *n* = 3.

To further verify the regulatory role in H/R injury, we analysed the effect of CHI3L1 knockdown and overexpression on IL‐1β, IL‐6 and MDA levels and SOD activity in IEC‐6 cells. IEC‐6 cells in the H/R+CHI3L1‐si‐1 and H/R+CHI3L1‐si‐2 groups had lower IL‐1β (all *P* < 0.0001), IL‐6 (all *P* < 0.0001) and MDA (all *P* < 0.0001) levels and higher SOD (all *P* < 0.001) activity than cells in the H/R+NC‐si group (Figure 7). IEC‐6 cells in the H/R+CHI3L1‐OE group had higher IL‐1β (*P* < 0.0001), IL‐6 (*P* = 0.0002) and MDA (*P* < 0.0001) levels and lower SOD activity (*P* = 0.002) than cells in the H/R+empty plasmid group (Figure 7). These results showed that CHI3L1 knockdown alleviated and CHI3L1 overexpression aggravated H/R‐induced inflammatory response and oxidative stress.

### 3.5 Effect of CHI3L1 on the PI3K/AKT pathway and the transcription factors NF‐κB and Nrf2

**FIGURE 8:**
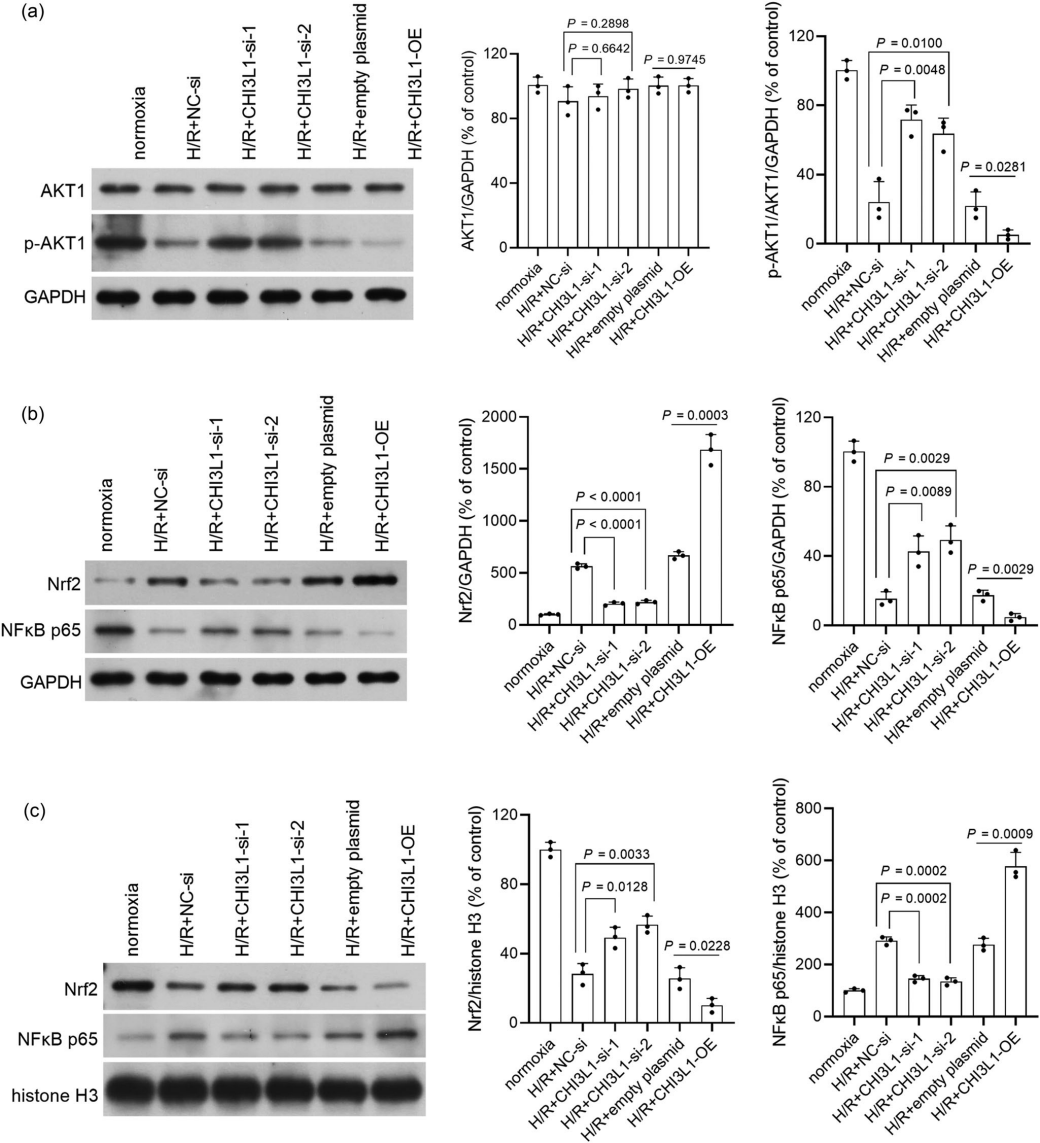
Effect of CHI3L1 knockdown and CHI3L1 overexpression on AKT1 phosphorylation and the distribution of NF‐κB p65 and Nrf2 in cytoplasm and nucleus. CHI3L1‐si‐1 and CHI3L1‐si‐2 were transfected into IEC‐6 cells to knock down CHI3L1. CHI3L1‐OE was transfected into IEC‐6 cells to overexpress CHI3L1. After transfection, IEC‐6 cells were cultured under hypoxia for 2 h and reoxygenated for 6 h (H/R). Cells cultured under normoxia were used as a control. Protein levels were measured using western blotting. (a) p‐AKT1 and AKT1 levels. (b) NF‐κB p65 and Nrf2 levels in the cytoplasm. GAPDH is the reference protein. (c) NF‐κB p65 and Nrf2 levels in the nucleus. Histone H3 is the reference protein. Right panels, bar graph of the relative expression level of each target protein. Three data points were overlaid on the bar graph. For all groups, *n* = 3.

To investigate the potential mechanism by which CHI3L1 regulates H/R injury, we analysed the impact of CHI3L1 knockdown and overexpression on the PI3K/AKT pathway and the distribution of transcription factors NF‐κB and Nrf2 in cytoplasm and nucleus. As shown in Figure 8a, p‐AKT1 levels were higher in H/R+CHI3L1‐si‐1 and H/R+CHI3L1‐si‐2 cells than in H/R+NC‐si cells and were lower in H/R+CHI3L1‐OE cells than in H/R+empty plasmid cells (all *P* < 0.05). The above results showed that CHI3L1 knockdown enhanced and CHI3L1 overexpression weakened the activation of the PI3K/AKT pathway. In addition, we found that the cytoplasmic NF‐κB p65 level was higher and the nuclear NF‐κB p65 level was lower in H/R+CHI3L1‐si‐1 and H/R+CHI3L1‐si‐2 cells than those in of H/R+NC‐si cells (all *P* < 0.01) (Figure 8b,c). In addition, cytoplasmic Nrf2 levels were lower and nuclear Nrf2 levels were higher in H/R+CHI3L1‐si‐1 and H/R+CHI3L1‐si‐2 cells than those in H/R + NC‐si cells (all *P* < 0.05) (Figure 8b,c). These results indicated that CHI3L1 knockdown promoted the nuclear translocation of Nrf2 and suppressed the nuclear translocation of NF‐κB, and that CHI3L1 overexpression suppressed the nuclear translocation of Nrf2 and promoted the nuclear translocation of NF‐κB (Figure 8b,c).

### 3.6 LY294002 partially restored the effect of CHI3L1 knockdown on IEC‐6 cells

**FIGURE 9:**
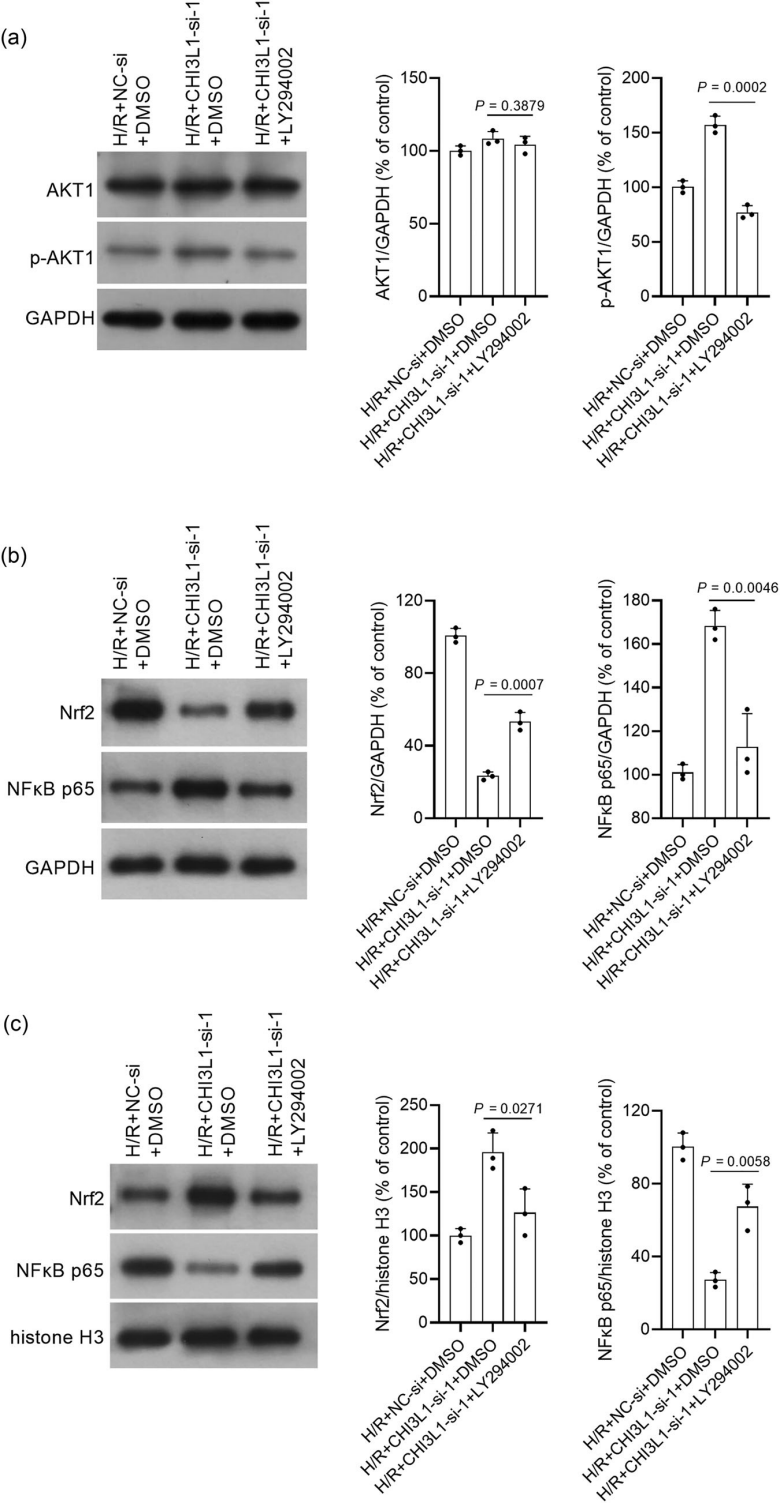
LY294002 blocked the effect of CHI3L1 knockdown on p‐AKT1, Nrf2 and NF‐κB p65 in IEC‐6 cells under H/R treatment. CHI3L1‐si‐1 was transfected into IEC‐6 cells to knock down CHI3L1 and NC‐si was transfected as a control. After transfection, IEC‐6 cells were cultured under hypoxia for 2 h and reoxygenated for 6 h (H/R) with DMSO or LY294002 treatment. (a) p‐AKT1 and AKT1 levels. (b) NF‐κB p65 and Nrf2 levels in the cytoplasm. GAPDH is the reference protein. (c) NF‐κB p65 and Nrf2 levels in the nucleus. Histone H3 is the reference protein. Right panels, bar graph of the relative expression level of each target protein. Three data points were overlaid on the bar graph. For all groups, *n* = 3.

**FIGURE 10:**
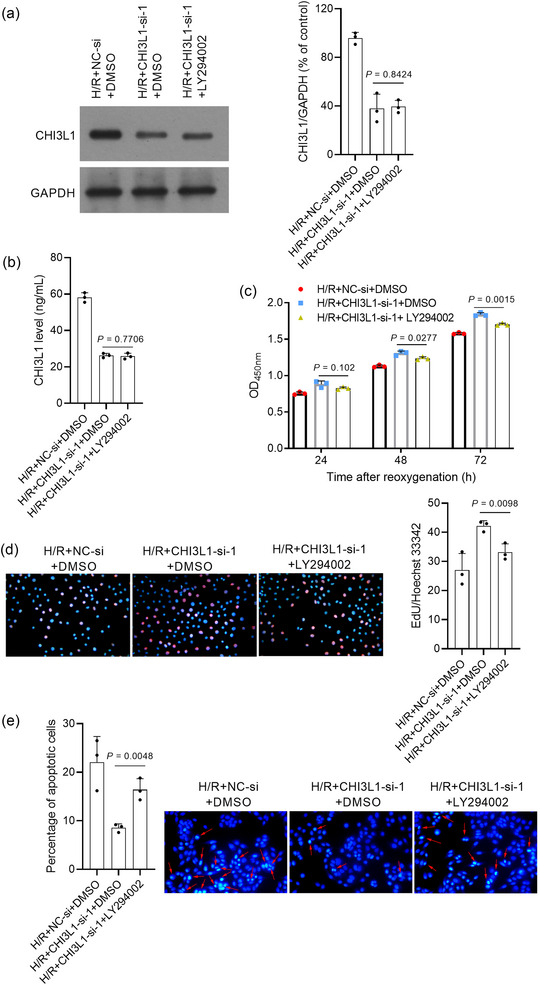
LY294002 blocked the effect of CHI3L1 knockdown on H/R‐induced proliferation inhibition and apoptosis. CHI3L1‐si‐1 was transfected into IEC‐6 cells to knock down CHI3L1 and NC‐si was transfected as a control. After transfection, IEC‐6 cells were cultured under hypoxia for 2 h and reoxygenated for 6 h (H/R) with DMSO or LY294002 treatment. (a) CHI3L1 level detected by western blot. Right panel, bar graph of the ratio of target protein and reference protein. (b) CHI3L1 level in culture medium measured by ELISA. (c) The OD_450 nm_ value of cells after continued culture for 24, 48 and 72 h after reoxygenation, detected by Cell Counting Kit‐8 assay. (d) EdU cell proliferation assay. Red is EdU‐positive cells; nuclei are blue. magnification: 20×. Right panel, bar graph of the percentage of EdU‐positive cells. (e) Hoechst 33258 staining. Nuclei were stained by Hoechst 33258 and appear blue; apoptotic cells appear white and are indicated using arrows. magnification: 20×. Left panel, bar graph of the percentage of apoptotic cells. Three data points were overlaid on the bar graph. For all groups, *n* = 3.

**FIGURE 11:**
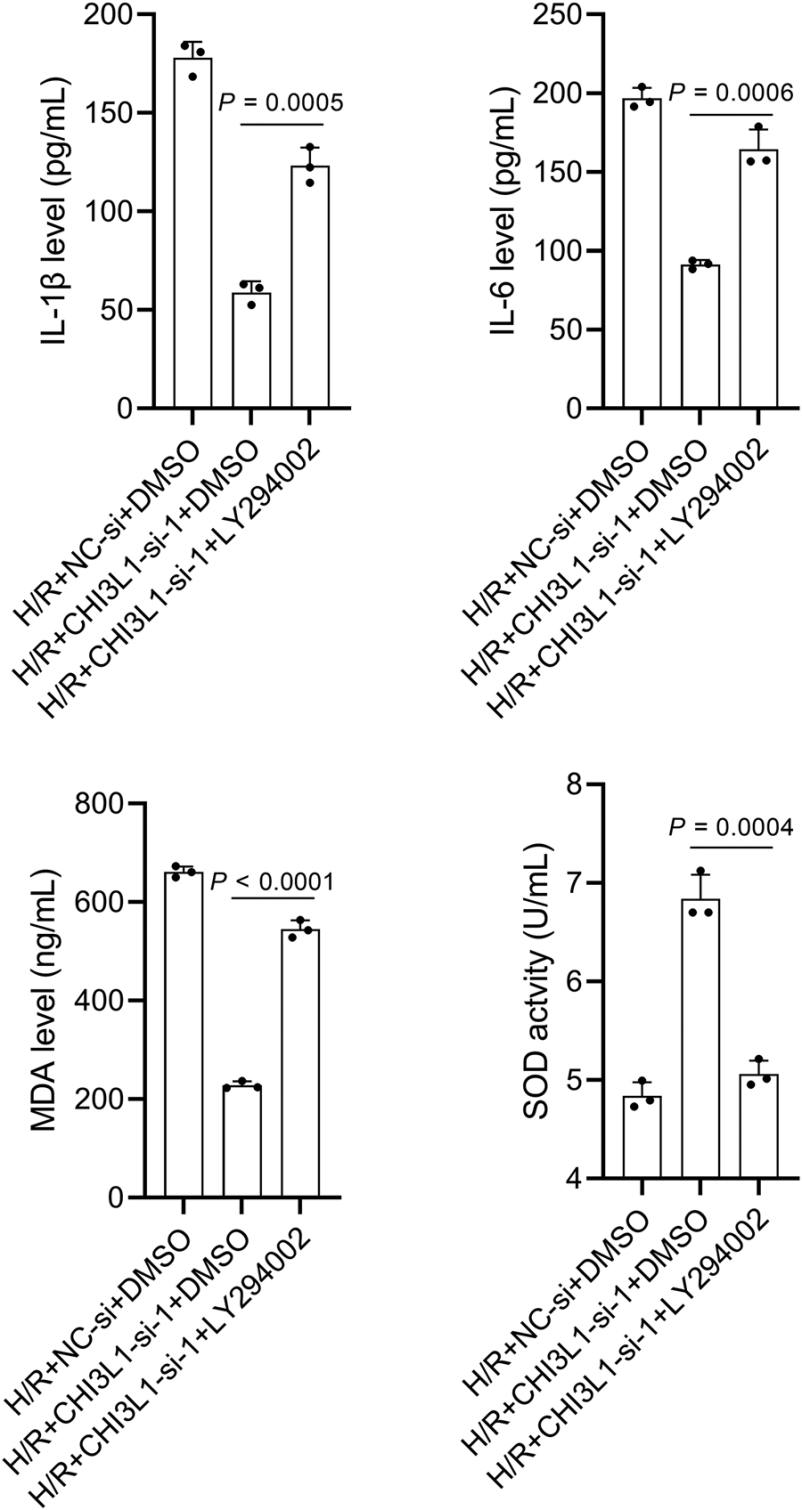
LY294002 blocked the effect of CHI3L1 knockdown on IL‐1β, IL‐6 and MDA levels and SOD activity in H/R‐treated IEC‐6 cells. CHI3L1‐si‐1 was transfected into IEC‐6 cells to knock down CHI3L1 and NC‐si was transfected as a control. After transfection, IEC‐6 cells were cultured under hypoxia for 2 h and reoxygenated for 6 h (H/R) with DMSO or LY294002 treatment. Three data points were overlaid on the bar graph. For all groups, *n* = 3.

To further verify whether CHI3L1 regulated H/R injury via the PI3K/AKT pathway, LY294002, a PI3K inhibitor, was used for subsequent rescue experiments. As shown in Figure 9a, p‐AKT1 levels were significantly decreased following LY294002 treatment (*P *= 0.0002) and were even lower than those in the H/R+NC‐si+DMSO group (*P *= 0.0082), suggesting that LY294002 treatment completely restored the effects of CHI3L1 knockdown on the PI3K/AKT pathway. The results in Figure 9b,c showed that LY294002 treatment partially restored the effects of CHI3L1 knockdown on the nuclear translocation of Nrf2 and NF‐κB (all *P* < 0.05). LY294002 did not impact the expression of CHI3L1 (ELISA: *P *= 0.7706; western blotting: *P *= 0.8424) (Figures 10a,b). Compared with cells in the H/R+CHI3L1‐si‐1+DMSO group, cells in the H/R+CHI3L1‐si‐1+LY294002 group had lower OD_450nm_ values at 48 h (*P *= 0.0277) and 72 h (*P *= 0.0015) after reoxygenation (Figure 10c) and EdU‐positive cells (*P *= 0.0098) (Figure 10d), higher apoptosis levels (*P *= 0.0048) (Figure 10e), higher levels of IL‐1β (*P *= 0.0005), IL‐6 (*P *= 0.0006) and MDA (*P *< 0.0001) (Figure 11), and lower SOD activity (*P *= 0.0004) (Figure 11). In addition, we observed obvious differences between the H/R+NC‐si+DMSO and H/R+CHI3L1‐si‐1+LY294002 groups (all *P* < 0.05), suggesting that LY294002 treatment partially restored the effects of CHI3L1 on cell proliferation, apoptosis, inflammation and oxidative stress.

## 4 DISCUSSION

CHI3L1 is a well‐characterized protein. It is involved in the inflammatory response and plays a role in macrophage polarization (Jingjing et al., 2017), T‐helper cell type 2 inflammatory response (Ahangari et al., 2015), the functional transformation of dendritic cells (Ge et al., 2023), and IL‐13‐induced inflammation as the ligand of IL‐13Rα2 (He et al., 2020a). In addition, CHI3L1 plays a role in the response to and coping with environmental changes such as hypoxia (Miao et al., 2020). The role of CHI3L1 in intestinal inflammation has also been studied; CHI3L1 levels were found to be significantly elevated in faecal samples from patients with ulcerative colitis (Aomatsu et al., 2011) and in the inflamed mucosa of patients with inflammatory bowel disease (Ning et al., 2019). CHI3L1 exacerbates intestinal inflammation and the development of inflammatory bowel disease (Mizoguchi & Mizoguchi, 2007; Tran et al., 2014). Intestinal I/R injury can also induce inflammatory responses, resulting in the release of numerous inflammatory factors. The inflammatory cytokines IL‐1β and tumour necrosis factor α (TNF‐α) promote the expression of CHI3L1 (Recklies et al., 2005). Therefore, we hypothesized that CHI3L1 plays a role in intestinal I/R injury.

In the present study, we found that intestinal I/R injury increased the CHI3L1 levels in the serum, ileum and duodenum. This aberrant expression profile suggests that CHI3L1 plays a regulatory role in the pathogenesis of intestinal I/R injury. To test this hypothesis, an H/R IEC‐6 model was constructed. The IEC‐6 cell line is commonly used to study small intestinal injury (Almoiliqy et al., 2020; Wang et al., 2021). Our results show that H/R enhances CHI3L1 expression in IEC‐6 cells. The in vitro expression profile of CHI3L1 was consistent with the in vivo expression profile. This suggests that CHI3L1 plays a regulatory role in the pathology of intestinal I/R. We found that H/R‐induced inhibition of proliferation and apoptosis was alleviated by CHI3L1 knockdown and aggravated by CHI3L1 overexpression. In addition, CHI3L1 knockdown alleviated and CHI3L1 overexpression aggravated the H/R‐induced inflammatory responses and oxidative stress. These results suggest that CHI3L1 may play a promising role in inducing intestinal I/R injury, and that inhibition of CHI3L1 may be a new way to weaken intestinal I/R injury. Although the role of CHI3L1 in intestinal I/R has not yet been reported, its role in I/R injury in the liver (Jin et al., 2023), brain (Im et al., 2020) and kidney (Montgomery et al., 2017) supports our conclusions.

The PI3K/AKT signalling pathway is an intracellular signal transduction pathway that promotes metabolism, cell proliferation, survival and angiogenesis in response to extracellular signals (Yu et al., 2022). Activation of the PI3K/AKT signalling pathway can protect against intestinal I/R by inhibiting inflammatory responses, oxidative stress and apoptosis (Deng & Zhou, 2023; Wang et al., 2022; Zou & Wang, 2022). Our present study also showed that CHI3L1 knockdown activated and CHI3L1 overexpression inactivated the PI3K/AKT signalling pathway during H/R in IEC‐6 cells. The regulatory relationship between CHI3L1 and the PI3K/AKT signalling pathway has also been reported by other researchers (Li et al., 2024; Rusak et al., 2016). CHI3L1 is a secretory protein (Rusak et al., 2016), so how does it affect the intracellular signal transduction pathway PI3K/AKT? The extracellular receptors and ligands of CHI3L1 include IL‐13Rα2, transmembrane protein 219, galectin 3, prostaglandin D2 receptor 2, CD44 and syndecan‐1 (Zhao et al., 2020). The interaction of CHI3L1 with these receptors and ligands can activate or inactivate downstream signalling pathways, including PI3K/AKT signalling. Future studies should identify the specific intracellular signals that interact with CHI3L1 during intestinal I/R injury.

The present study suggests that CHI3L1 plays a role in intestinal I/R injury via the PI3K/AKT signalling pathway. Molecular regulatory networks are highly complex; therefore, PI3K/AKT may not be the only downstream signalling pathway in which CHI3L1 exerts its function. Our results showed that the PI3K inhibitor LY294002 completely restored the effects of CHI3L1 knockdown on the PI3K/AKT pathway but partially restored the effect of CHI3L1 knockdown on H/R‐induced inhibition of proliferation, apoptosis, inflammatory response and oxidative stress. Thus, we speculated that CHI3L1 may play a role in inducing intestinal I/R injury by inhibiting the PI3K/AKT signalling pathway, but this pathway may not be the sole downstream signalling pathway through which CHI3L1 exerts its function.

To further elucidate the mechanism of the CHI3L1–PI3K–AKT axis in detail, we analysed its effect on the nuclear translocation of Nrf2 and NF‐κB, which are downstream signals of AKT (He et al., 2020b; Mullonkal & Toledo‐Pereyra, 2007). NF‐κB is an essential transcription factor that drives the transcription of inflammation‐related genes, such as those for IL‐1β, IL‐6 and TNF‐α (Barnabei et al., 2021). Nrf2 is a key transcription factor that prevents oxidative stress by inducing the expression of numerous genes encoding antioxidant enzymes (He et al., 2020b). In addition, it is reported that Nrf2 and NF‐κB signalling pathways are involved in intestinal I/R injury (Li, Wang, et al., 2021; Zou & Wang, 2022). Our findings indicate that CHI3L1 influences the nuclear translocation of Nrf2 and NF‐κB, which can be blocked by LY294002 after CHI3L1 knockdown. These results indicate that CHI3L1 may play its role in the nuclear translocation of Nrf2 and NF‐κB through the PI3K/AKT signalling pathway. Given the complexity of signalling pathway regulation, it is possible that the upstream and downstream components of Nrf2 and NF‐κB are involved in additional signalling pathways. Numerous studies will be required to fully elucidate the upstream and downstream regulatory mechanisms of Nrf2 and NF‐κB.

In conclusion, CHI3L1 expression was induced during intestinal I/R injury and CHI3L1 overexpression aggravated H/R injury in IEC‐6 cells by inhibiting the PI3K/AKT signalling pathway. The inhibition of CHI3L1 may be an effective way to alleviate intestinal I/R injury. The current investigation is preliminary. Further research employing intestine‐specific CHI3L1 knockout mice and intestinal stem cells is necessary to better understand the functional and molecular mechanisms of CHI3L1 in the regulation of intestinal H/R injury.

## AUTHOR CONTRIBUTIONS

Lei Mi and Jie Jin designed the experiments, performed animal‐related experiments, ELISA, western blotting, cell culture, cell function experiments, and data analysis. Yingying Zhang performed the immunohistochemistry and hematoxylin and eosin staining. Ming Chen, JianLi Cui, and Rui Chen participated in western blotting and ELISA. Xiao Zheng and Changqing Jing designed the experiments, oversaw their performance, and revised the manuscript. All authors have read and approved the final version of this manuscript and agree to be accountable for all aspects of the work, ensuring that questions related to the accuracy or integrity of any part of the work are appropriately investigated and resolved. All persons designated as authors qualify for authorship and all those who qualify for authorship are listed.

## CONFLICT OF INTEREST

The authors declare no conflicts of interest.

## FUNDING INFORMATION

No funding was received for this work.

## Data Availability

Data are available via request to the corresponding author
